# Multi-Level Targeting of the Phosphatidylinositol-3-Kinase Pathway in Non-Small Cell Lung Cancer Cells

**DOI:** 10.1371/journal.pone.0031331

**Published:** 2012-02-15

**Authors:** Christopher R. Zito, Lucia B. Jilaveanu, Valsamo Anagnostou, David Rimm, Gerold Bepler, Sauveur-Michel Maira, Wolfgang Hackl, Robert Camp, Harriet M. Kluger, Herta H. Chao

**Affiliations:** 1 Yale University School of Medicine & Yale Comprehensive Cancer, New Haven, Connecticut, United States of America; 2 Medical Service, VA Connecticut Healthcare System, West Haven, Connecticut, United States of America; 3 Karmanos Cancer Institute, Detroit, Michigan, United States of America; 4 Novartis Institutes for Biomedical Research, Cambridge, Massachusetts, United States of America; Univesity of Texas Southwestern Medical Center at Dallas, United States of America

## Abstract

**Introduction:**

We assessed expression of p85 and p110α PI3K subunits in non-small cell lung cancer (NSCLC) specimens and the association with mTOR expression, and studied effects of targeting the PI3K/AKT/mTOR pathway in NSCLC cell lines.

**Methods:**

Using Automated Quantitative Analysis we quantified expression of PI3K subunits in two cohorts of 190 and 168 NSCLC specimens and correlated it with mTOR expression. We studied effects of two PI3K inhibitors, LY294002 and NVP-BKM120, alone and in combination with rapamycin in 6 NSCLC cell lines. We assessed activity of a dual PI3K/mTOR inhibitor, NVP-BEZ235 alone and with an EGFR inhibitor.

**Results:**

p85 and p110α tend to be co-expressed (p<0.001); p85 expression was higher in adenocarcinomas than squamous cell carcinomas. High p85 expression was associated with advanced stage and poor survival. p110α expression correlated with mTOR (ρ = 0.276). In six NSCLC cell lines, addition of rapamycin to LY294002 or NVP-BKM120 was synergistic. Even very low rapamycin concentrations (1 nM) resulted in sensitization to PI3K inhibitors. NVP-BEZ235 was highly active in NSCLC cell lines with IC_50_s in the nanomolar range and resultant down-regulation of pAKT and pP70S6K. Adding Erlotinib to NVP-BEZ235 resulted in synergistic growth inhibition.

**Conclusions:**

The association between PI3K expression, advanced stage and survival in NSCLC suggests that it might be a valuable drug target. Concurrent inhibition of PI3K and mTOR is synergistic *in vitro*, and a dual PI3K/mTOR inhibitor was highly active. Adding EGFR inhibition resulted in further growth inhibition. Targeting the PI3K/AKT/mTOR pathway at multiple levels should be tested in clinical trials for NSCLC.

## Introduction

Lung cancer is the leading cause of cancer-related death worldwide. In the United States alone, about 222,520 new cases were diagnosed and about 157,300 deaths occurred due to lung cancer in 2010 [1]. Non-small cell lung cancer (NSCLC) represents about 80% of all lung cancer. The most common histologies are adenocarcinoma, squamous cell carcinoma, and large cell carcinoma. The majority of NSCLC patients are diagnosed at advanced stage where chemotherapy may improve survival and palliation of symptoms. However, conventional chemotherapy provides no cure for advanced NSCLC and has reached a plateau in efficacy with a median survival of 8–10 months [2]. The addition of new targeted therapies such as bevacizumab and cetuximab to conventional chemotherapy has improved the median survival to about 12 months in patients with good performance status [3], [4]. Novel therapeutic approaches are urgently needed for this common disease.

The phosphatidylinositol-3-kinase (PI3K)/AKT/mTOR signaling pathway impacts many aspects of cell growth and survival [5]. Alterations of components in the PI3K/AKT/mTOR pathway can occur at many levels and result in constitutive activation of this pathway and malignant transformation.

The PI3Ks are a family of enzymes that phosphorylate phosphatidylinositol biphosphate to phosphatidylinositol triphosphate. PI3Ks are most often activated by receptor tyrosine kinase (RTK) signaling such as through EGFR, IGF1-R and HER2/neu [6]–[9]. There are three classes of PI3Ks [10], [11]. Class I_A_ PI3K is the most widely implicated type in cancer and will be referred to as “PI3K” in the remainder of this manuscript. PI3K is a heterodimer consisting of a p85 regulatory and a p110 catalytic subunit.

Phosphatidylinositol triphosphate mediates the activation of AKT [11]. AKT, in turn, activates many cellular proteins involved in protein synthesis, cell growth and survival including mTOR [11]–[13]. mTOR regulates translation by phosphorylating components of the protein synthesis machinery, including the ribosomal protein S6 kinases (p70^S6K^) and 4E-binding protein (4E-BP). Phosphorylation of 4E-BP leads to the release of the translation initiation factor eIF4E which has been demonstrated to exhibit transforming and anti-apoptotic activites *in vitro* [13], [14]. PTEN reverses PI3K signaling by dephosphorylating phosphatidylinositol triphosphate [15].

In NSCLC, PI3K/AKT/mTOR signaling is frequently deregulated due to mutations affecting one of its upstream regulators, the EGFR receptor, and other components within the pathway [16]. mTOR pathway components were found to be mutated in 17 genes and in more than 30% of tumors of 188 lung adenocarcinomas in which exome sequencing was performed [16]. Increases in gene copy number of *PIK3CA*, the gene encoding p110α, and changes in phosphorylated AKT (pAKT) expression have been described in premalignant bronchial epithelial cells and NSCLC [17]–[22]. While mutations in *PIK3CA* are relatively infrequent in lung cancer, *PIK3CA* copy number gain has been reported in 33.1% of squamous cell lung cancer and in 6.2% adeno lung cancer in one large series [23]. PI3K signaling has been shown to mediate bronchioalveolar stem cell expansion initiated by oncogenic *K-RAS* [24]. Tumor associated mutations of p110α are oncogenic *in vivo* in a mouse model of NSCLC [25]. Overexpression of p85 and p110 α has been demonstrated to correlate with poor differentiation of primary lung cancers in a cohort that included 73 cases of NSCLC [26]. Our group has previously studied the expression of mTOR in NSCLC cohorts and found an association with improved outcome [27].

Inhibition of PI3K/AKT/mTOR signaling through pharmacologic and genetic approaches induces antiproliferative effects on certain NSCLC cell lines [17]–[21] and in lung cancer mouse models [25], [28]. A number of PI3K inhibitors are available for preclinical research. Older compounds like LY294002 or wortmannin have anti-tumor activity in preclinical models, but their poor solubility, narrow therapeutic index and crossover inhibition of other kinases have limited their clinical application. Newer PI3K inhibitors have entered early phase clinical trials, and activity of these agents should be assessed in diseases requiring new approaches, such as NSCLC.

The purpose of our study was to characterize the expression of p85 and p110 α subunits of Class I_A_ PI3K in two large independents cohorts of NSCLC specimens and to assess the association with clinical and pathological variables including previously published mTOR expression. To obtain more precise, objective expression measures, we used a newly developed method of automated, quantitative analysis (AQUA) of tissue microarrays [29]. As redundant activators of the PI3K/AKT signaling pathway and negative feedback loops [5] limit the efficacy of single agent therapies, our next purpose was to study the effects of targeting the PI3K/AKT signaling pathway at multiple levels in NSCLC cell lines. We found that higher expression of p85 correlated with poor survival and advanced stage. Expression of p110α correlated with that of mTOR. Concurrent inhibition of PI3K and mTOR resulted in synergistic growth suppression. Adding EGFR inhibition further enhanced the growth-inhibitory effects of a dual PI3K/mTOR inhibitor.

## Materials and Methods

### Tissue Microarray (TMA) Construction

A NSCLC cohort was obtained from the H. Lee Moffitt Cancer Center (Tampa, FL). The Moffitt Cancer Center cohort (MTMA) contains cores from primary NSCLC tumors of patients diagnosed between 1991 and 2001. Follow-up time ranged between 0.8 months and 146.4 months, mean follow-up time of 52.3 months. Age at diagnosis ranged from 40.8 to 84.4 (mean age 69 years). The cohort included 54.5% males and 45.5% females.

The Yale University cohort (YTMA) was constructed from paraffin-embedded, formalin-fixed tissue blocks obtained from the Yale University Department of Pathology Archives. The specimens were resected between 1995 and 2003, with a follow-up range between 0.1 months and 182.25 months, and a mean follow-up time of 41 months. Age at diagnosis ranged from 21 to 90 (mean age 65 years). The cohort included 51% males and 49% females.

TMAs were constructed as previously described [27]. Two 0.6 mm cores were obtained from different, representative areas of each primary NSCLC specimen and spaced 0.8 mm apart on glass slides. Cell line pellets consisting of SW480, HT29, A431, MB435, MCF7, BT474, and SKBR3 were used as controls and were embedded in the array, as previously described [30]. The cohorts for MTMA and YTMA were collected with approval of the institutional review boards and have been used in prior publications [27], [31].

### Immunofluorescent staining

TMA slides were stained for each of the two target markers, PI3K p85 and p110α subunits. Staining was performed for AQUA as described previously [29]–[31]. Slides were incubated with the primary antibody diluted in Tris-buffered saline containing 0.3% bovine serum albumin at 4°C. Primary antibodies used for the respective incubations were mouse monoclonal anti-human PI3K p85, clone 4/PI3-Kinase (Becton Dickinson, Franklin Lakes, NJ) or rabbit anti-human PI3K p110α clone C73F8 (Cell Signaling Technology, Danvers, MA), at 1∶200 or 1∶50 dilutions, respectively. Either goat anti-mouse or goat anti-rabbit horseradish peroxidase-decorated polymer backbone (Envision, Dako North America, Carpinteria, CA) was utilized to visualize the target protein. To create a tumor mask, slides were simultaneously incubated with either mouse or rabbit anti-cytokeratin at 1∶100. For visualization of cytokeratin staining a goat anti-mouse or anti-rabbit IgG conjugated to Alexa 546 (Molecular Probes, Inc.) at 1∶200 was utilized. The target marker was visualized with Cy5-tyramide (NED Life Science Products). Coverslips were mounted with ProLong Gold reagent with 4′,6-diamidino-2-phenylindole (DAPI) (Invitrogen).

### Automated Image Acquisition and Analysis (AQUA)

Images were analyzed using algorithms that have been described [29]. Tumor was distinguished from stromal elements by cytokeratin signal. Coalescence of cytokeratin at the cell surface was used to localize cell membrane/cytoplasmic compartment within the tumor mask, and DAPI was used to identify the nuclear compartment within the tumor mask. Targets were visualized with Cy5; this wavelength is used for target labeling because it is outside the range of tissue autofluorescence. Multiple monochromatic, high-resolution (1,024×1,024 pixel, 0.5-µm) grayscale images were obtained for each histospot using the 10× objective of an Olympus AX-51 epifluorescence microscope with automated microscope stage and digital image acquisition driven by a custom program and macrobased interfaces with IPLabs software (Scanalytics, Inc.). Images for each histospot were individually reviewed. Two images were captured for each histospot and for each fluorescent channel, DAPI, Alexa-546, and Cy5; one image in the plane of focus and one 8 ìm below it. The compartmentalization and quantification of the target protein signal within each pre-defined compartment for each histospot was performed as follows. First, the Alexa-546 signal representing cytokeratin staining was utilized to generate an epithelial cell mask that excludes all other stromal elements. This signal is binary gated in order to identify whether a pixel is within the tumor mask (on) or not (off); all white pixels are part of that mask and all black pixels are not part of this compartment. Similarly, the nuclear compartment is defined as pixels that demonstrate DAPI staining within the plane of focus and within the region defined by the tumor mask. The DAPI image is also binarized to generate a mask of all nuclei within the sample by subtracting out overlapping pixels with the cytoplasmic mask; all white pixels are part of this mask while all black pixels are not. To ensure that only the target signal from the tumor and not the surrounding elements is analyzed, the RESA/PLACE algorithms were utilized. The RESA algorithm provides an adaptive thresholding system. In general, formalin-fixed tissues can exhibit autofluorescence and sometimes analysis can give multiple background peaks. The RESA algorithm establishes the predominant peak and then sets a binary mask threshold at a slightly higher intensity level. RESA eliminates all out-of-focus information by subtracting a percentage of the out-of-focus image from the in-focus image, based on a pixel-by-pixel analysis of the two images. This eventually allows more accurate assignment of pixels of adjacent compartments. Finally, we utilize the PLACE algorithm to assign each pixel of each image to a specific subcellular compartment. All pixels that cannot be accurately assigned to a compartment with a degree of confidence of 95% are ultimately excluded. Additionally, all pixels for which intensities are too similar in the nuclear and membrane compartments and therefore cannot be accurately assigned are also excluded. PI3K expression was automatically determined from Cy5 channel images to obtain relative pixel intensity for the signal emanating from the plane of focus. First, each pixel is assigned to a subcellular compartment or is excluded as described above. An AQUA score for any sub-cellular compartments is usually calculated as the average AQUA score from each of the individual pixels included in the selected compartment; the target signal (p85 or P110α) from the pixels within the cytoplasm was normalized to the area of tumor mask and scored on a scale of 0–255 (the AQUA score). Histospots were excluded if the tumor mask represented less than 5% of the total histospot area.

### Statistical Analysis

JMP version 5.0 software was used (SAS Institute, Cary, NC). The prognostic significance of parameters was assessed using the Cox proportional hazards model with progression free survival and overall survival as an end point. Univariate survival analyses were depicted using the Kaplan-Meier method. The association between continuous AQUA scores and other clinical/pathological parameters was assessed by analysis of variance. All reported p-values are based on two-sided significance testing. ANOVA and t-test were used to compare continuous measurements.

### Cell Lines and Western Blots

Squamous carcinoma cell line H2170 and adenocarcinoma cell lines H1650, HCC2935 and HCC827 were kindly provided by Drs. John Minna and Michael Peyton (Southwestern University). Squamous carcinoma cell lines SK-MES-1 and SW900 were obtained from American Type Tissue Culture. The characteristics of each cell line including mutational status of *PIK3CA*, *K-RAS* and *EGFR* are shown in **Table S1**. The adenocarcinoma cell lines H1650, HCC2935 and HCC827 have wild-type *PIK3CA* and *K-RAS* but mutant *EGFR*. All squamous carcinoma cell lines SK-MES-1, H2170 and SW900 have wild-type *EGFR* and wild-type *PIK3CA*. SK-MES-1 and H2170 have wild-type *K-RAS*. SW900 has mutant *K-RAS*. We were interested in comparing the effects of PI3K inhibition in adenocarcinoma to squamous carcinoma cell lines and comparing with *EGFR* mutant to *EGFR* wild-type cell lines. All human lung cancer cell lines were cultured under standard conditions (37°C in 5% CO_2_ atmosphere) and grown in RPMI (Gibco™, Invitrogen Corp, Grand Island, NY) supplemented with 10% FBS. HBE135-E6E7 cell line, a non-transformed bronchoalveolar cell line, was purchased from the American Type Culture Collection (Manassas,VA) and grown in Keratinocyte-Serum Free medium supplemented with 5 ng/ml human recombinant EGF, 0.05 mg/ml bovine pituitary extract, 0.005 mg/ml insulin and 500 ng/ml hydrocortisone.

Protein concentrations of cell lysates were calculated by the BCA (Bicinchoninic Acid) assay (Pierce Biotechnology, Rockford, IL). Proteins (50 µg) were diluted in a sample buffer [2.5% SDS, 10% glycerol, 5% β-mercapto-ethanol, 50 mM Tris (pH = 6.8) and 0.1% bromophenol blue] and subjected to sodium dodecyl sulfate-polyacrylamide gel electrophoresis (SDS-PAGE). Western blotting was performed by standard methods using the following primary rabbit anti-human antibodies: phosporylated AKT (Ser^473^), phosphorylated p70S6K (Thr^389^), PI3K p110α, phosphorylated S6 Ribosomal Protein (Ser^235/236^), PARP (Cell Signaling Technologies, Danvers, MA) at 1∶1000, and mouse monoclonal anti-human PI3K p85 or anti-caspase-2 (Becton Dickinson, Franklin Lakes, NJ) at 1∶1000. A monoclonal mouse β-Actin (A2066 or A5441, SIGMA, 1∶200 or 1∶5000 respectively) was utilized as a control to standardize sample loading. Detection of proteins was done with peroxidase-conjugated anti-mouse or anti-rabbit IgG secondary antibodies (1∶5000, Jackson ImmunoResearch Laboratories) and ECL (PerkinElmer).

### Antineoplastic Agents and Cell Viability Assays

For cell viability assays, 1×10^3^ cells were plated in triplicate in a 96 well microtiter plate (BD Bioscience) and allowed to grow for 24 hours to an approximate confluence of 30%. For drug inhibition studies, NVP-BEZ235 and NVP-BKM120 (Novartis Pharmaceuticals, Basel, Switzerland) and LY294002 (LC Laboratories, Woburn MA) were used to treat lung cells at various concentrations. For combination experiments, the mTORC1 inhibitor, rapamycin and EGFR inhibitor erlotinib (LC Laboratories) were utilized.

Cell viability was evaluated at 72 hours using the CellTiter-Glo™ Luminescent Cell Viability Assay, according to the manufacturer's instructions (Promega, USA); an equal volume of the Cell Titer-Glo™ reagent was added to the wells and after a 10 minute incubation, luminescence was recorded using a Victor™X multilabel plate reader (Perkin Elmer). The IC_50_ values were determined by the XLfit software (MathIQ version 2.2.2, IDBS).

### Synergistic Drug Effect Analysis

The Chou and Talalay combination index analysis method was utilized to analyze results from combinatory drug experiments [32]. NVP-BKM120 and LY294002 were used in combination with the mTOR inhibitor, rapamycin. NVP-BEZ235 was combined with erlotinib, at their respective single drug IC_50_ or IC_25_ concentrations. The IC_50_ and IC _25_ values of each drug were first obtained from single-drug viability assays and then utilized to design drug combination experiments. The level of synergism was assessed over a wide range of drug concentrations, focusing on concentrations below or at the IC_50_ for either drug alone. Our results were analyzed for synergistic, additive, or antagonistic effects using CalcuSyn software (Biosoft, Ferguson, Missouri, USA). Synergistic effect is indicated by a Combination Index (CI) of less than 0.9, additive effect by a CI between 0.9 and 1.1, and antagonistic effect by CI greater than 1.1.

## Results

### Expression of PI3K p85 and p110á subunits in human lung cancer specimens

**Figure 1:**
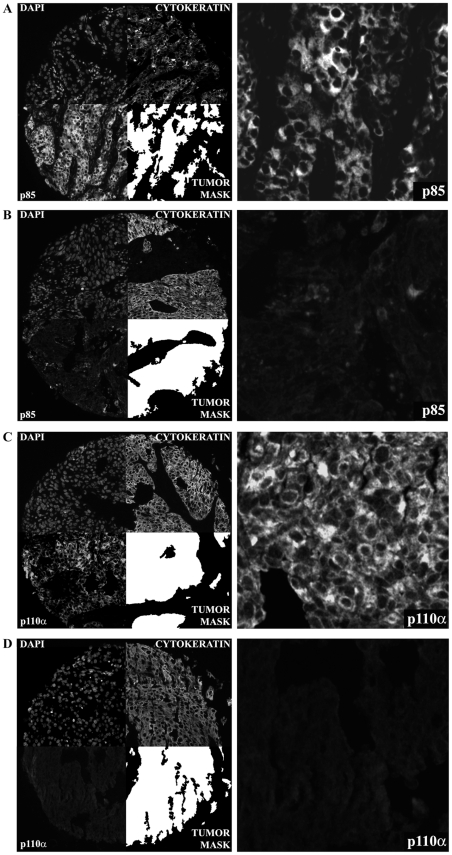
Examples of positive and negative staining of histospots for the p85 (Panel A and B, respectively) and p110α (Panel C and D, respectively) subunits. Anti-PI3K conjugated to Cy5 is used to measure PI3K subunit levels (lower left quadrants, magnification ×10). Anti-cytokeratin conjugated to Cy3 is used to identify tumor cells within the histospot (upper right quadrants). Holes are filled in to create a tumor mask (right lower quadrants). DAPI is used to identify nuclei (upper left quadrants). The nuclear compartment within the tumor mask is then subtracted from the mask to create a cytoplasmic compartment (not shown). The right panels are 40× magnifications of PI3K p85 and p110α subunit positive and negative staining, respectively.

For the MTMA, 166 and 190 specimens were fully assessable with regard to PI3K p85 and p110α subunit expression, respectively. PI3K did not show significant nuclear staining for either subunit, and we therefore analyzed only the cytoplasmic compartment. Staining patterns within the tumor mask within a histospot were highly homogenous for both subunits. AQUA scores ranged from 7.12 to 127.04 (mean, 37.68; median, 33.47) for the p85 subunit, and from 5.43 to 138.56 (mean, 24.35; median, 20.26) for the p110α subunit. An example of AQUA staining of histospots for p85 and p110α expression is shown in **Figure 1** **and Table S2**. Histospots were deemed un-interpretable if they had insufficient tumor cells, loss of tissue in the spot, or an abundance of necrotic tissue. Specimens with less than 5% tumor area per spot were not included in the AQUA analysis.

**Figure 2:**
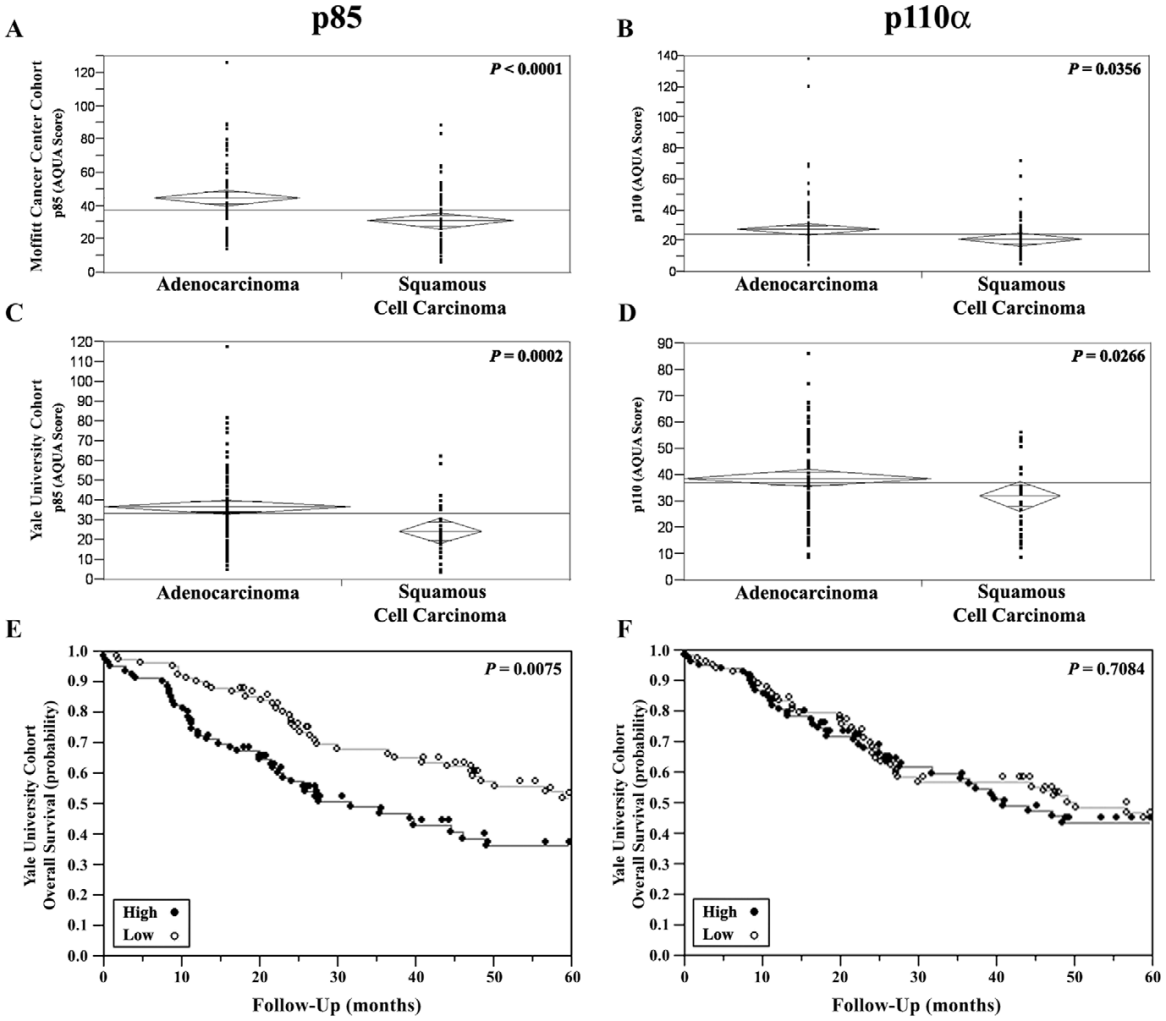
Unpaired t-tests showing the association between PI3K levels in NSCLC specimens and histological subtypes. Significantly higher expression of PI3K p85 subunit is seen in adenocarcinoma tumors, in the Moffitt Cancer Cohort and the Yale University Cohort, respectively **(Panel A & C)**. Significantly higher expression of PI3K p110α subunit in adenocarcinoma tumors is seen in the Moffitt Cancer Cohort and the Yale University Cohort, respectively **(Panel B & D)**. Kaplan Meier curves showing an association between PI3K subunit expression and decreased survival for p85 **(Panel E)** but not for p110α **(Panel F)**.

We assessed the associations between PI3K subunit expression and histologic subtype, by unpaired t-tests. Expression of both the p85 and the p110α subunits were significantly higher in adenocarcinomas than in squamous cell carcinomas (*P*<0.0001 for p85 and *P* = 0.0356 for p110α), as shown in **Figure 2** **, panel A & B**.

To validate the association between the PI3K subunits and adenocarcinoma, the YTMA was employed. As opposed to the MTMA which was primarily stage I (A and B) lung cancer (92%), the YTMA included 55% stage I, and 45% stage II–IV (17%, 19% and 9% stage II, III, IV respectively) lung cancer. For the YTMA PI3K p85 and p110α subunit expression was interpretable for 163 and 168 specimens, respectively. The AQUA scores for this cohort ranged from 4.18 to 120.73 (mean, 35.31; median, 32.70) for the p85 subunit, and from 9.17 to 86.91 (mean, 38.12; median, 35.17) for the p110α subunit. Unpaired t-test confirmed the findings from the MTMA; expression of the p85 subunit and the p110α subunit were significantly higher in adenocarcinomas than in squamous cell carcinomas (*P*<0.0002 for p85 and *P* = 0.0266 for p110α), as shown in **Figure 2** **, panel C & D**. High p85 and p110α subunit expression correlated with advanced disease stage of disease (*P* = 0.0075, *P* = 0.0093, respectively), but not with age and gender (not shown).

By Cox univariate survival analyses of continuous AQUA scores, we found that high p85 expression correlated with decreased survival (*P* = 0.0198). AQUA provides continuous output scores, rather than categories of “high” or “low”, as determined by pathologists interpreting standard immunohistochemistry. To visualize the association between continuous PI3K scores and survival, AQUA scores were dichotomized by the median, reflecting the use of routine statistical divisions in the absence of underlying justification for division of expression. Kaplan-Meier survival curves were generated for PI3K p85 subunit expression and survival and *P*-values were obtained by the Mantel-Cox log-rank method. As shown in **Figure 2** **, panel E & F**, we found a significant association between high p85 expression and poor survival (*P* = 0.0075). No association was found between p110α expression and survival.

On multivariate analysis p85 expression did not retain its independent predictive value, presumably due to its association with histologic subtype and stage. The only variable associated with survival by multivariate analysis was Stage (*P* = 0.0000). No such association between p110α subunit expression and survival was noted.

### Coexpression of PI3K p85 or p110á with mTOR in human lung tumors

The association between the previously published mTOR expression [27] and p85 and p110α subunits of PI3K was assessed. Using the Spearman rank correlation test, mTOR expression was associated with p110α expression (ρ = 0.276, *p* = 0.0006), while no association was found between levels of mTOR and p85 subunit.

### *In vitro* activity of NVP-BKM120 and LY294002 in lung cell lines

Due to the association between PI3K and advanced stage and poor survival, the *in vitro* activity of PI3K inhibitors was studied in six human lung cancer cell lines. Two PI3K inhibitors were utilized; we studied NVP-BKM-120, a clinical quality PI3K inhibitor being developed by Novartis, and LY294002, a commercially available compound that has been widely used to study PI3K inhibition in the preclinical setting. Cells were treated with either NVP-BKM120 at concentrations ranging from 0.01 nM to 8,200 nM or LY294002 at concentrations ranging from 6.4 nM to 20,000 nM. All experiments were done in triplicate. After 72 hours incubation, cell viability was evaluated, and the IC_50_ was calculated for each cell line, and averaged for repeat experiments. As shown in **Table 1**, the IC_50_ for NVP-BKM120 and LY294002 inhibition ranged from 716 nM to 1265 nM and 4123 nM to 11925 nM, respectively.

**Table 1:** IC_50_ of NVP-BKM120, LY294002, and NVP-BEZ235 in a panel of six lung cancer cell lines.

Histological Subtype	Cell Line	IC50's (nM)		
		NVP-BKM120	LY294002	NVP-BEZ235
**Squamous Cell Carcinoma**	**SK-MES**	1091	4123	36
	**H2170**	821	9733	22
	**SW900**	1128	11652	50
**Adenocarcinoma**	**H1650**	1248	10850	23
	**HCC2935**	716	9463	9
	**HCC827**	1265	11925	130

To test whether growth inhibition inflicted by NVP-BKM120 and LY294002 are specific or at least enhanced in malignant compared to normal cells, we used an immortalized, non-transformed bronchoaveolar cell line, HBE135-E6E7. These cells were derived from normal bronchial epithelium taken from a man undergoing lobectomy for squamous cell carcinoma [33]. NVP-BKM120 and LY294002 had little effect on the normal bronchoaveolar cells at concentrations approximately 10 and 5 fold the average IC_50_ of these two drugs, respectively, in NSCLC cells. 13% growth inhibition was seen at 10 µM NVP-BKM120 but an IC_50_ could not be reached at higher concentrations, and a mere 25% growth inhibition was obtained with 50 µM LY294002 treatment.

Given that the expression levels of drug targets sometimes predict drug sensitivity, we studied the association between the degree of sensitivity/resistance to NVP-BKM120 and LY294002 and PI3K levels. We assessed the two PI3K subunits and total and phosphorylated AKT by immunoblotting. No clear association was found between pretreatment levels of PI3K (p85 or p110α) or total and phosphorylated AKT and sensitivity/resistance to NVP-BKM120 or LY294002 (**Figure S1**).

### Synergism between PI3K and mTOR inhibitors

**Figure 3:**
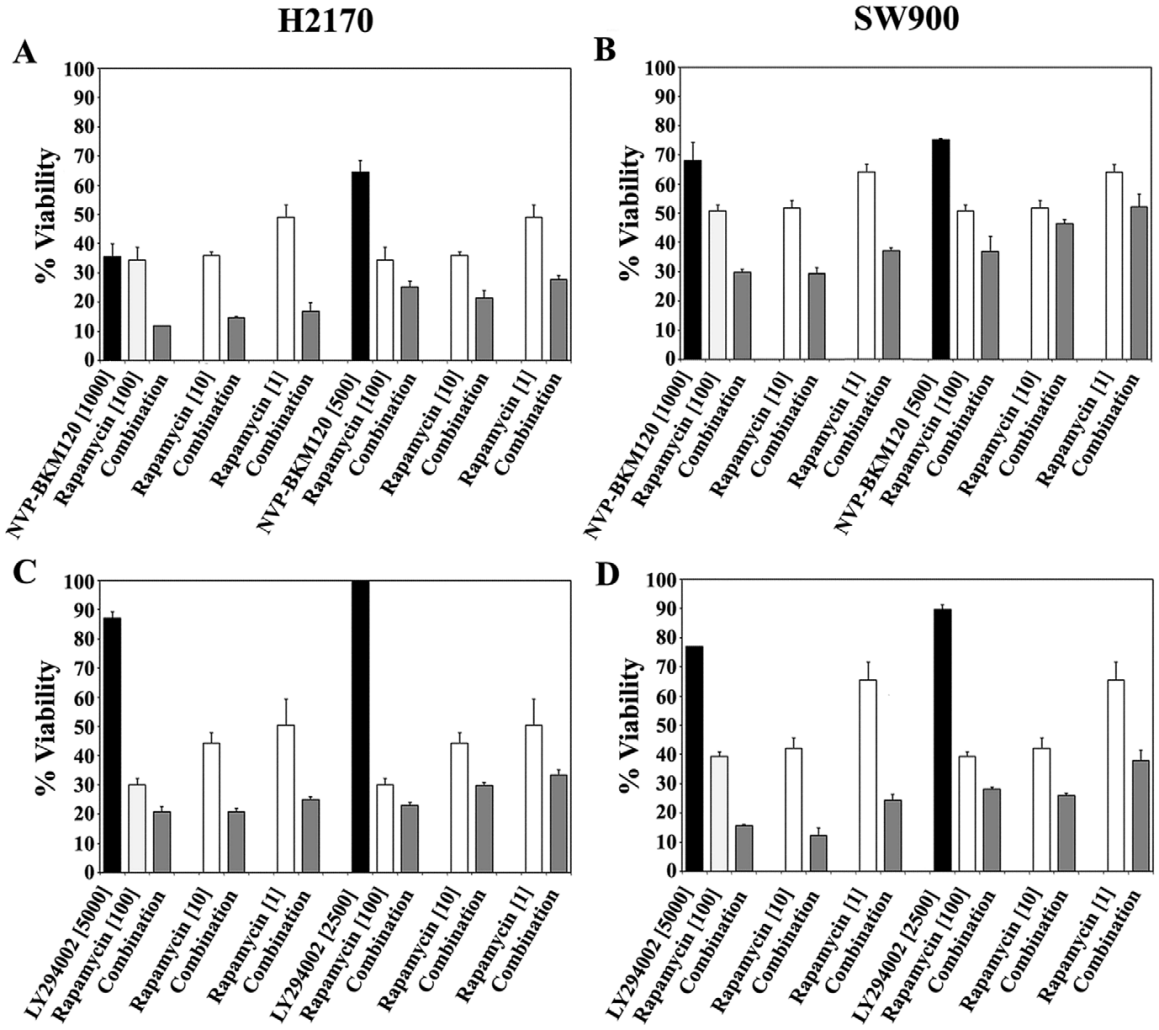
Combinations of either NVP-BKM120 and rapamycin or LY294002 and rapamycin in H2170 (left) and SW900 (right) lung cancer cell lines. Cells were treated with descending nanomolar concentrations of the PI3K inhibitor NVP-BKM120 or LY294002, alone or combined with 1 nM, 10 nM or 100 nM rapamycin for 72 hours and viability was measured with the Cell Titer Glo assay. The bars show viability as a percentage of viable cells relative to untreated cells. Three separate experiments were performed and each condition was measured in three replicate wells.

Resistance to PI3K inhibition has been noted in different diseases and attributed to numerous mechanisms. Constitutive PI3K pathway activation could result from AKT activation by mTOR-C2 or mTOR activation by MAP kinase pathway members despite specific PI3K drug inhibition. Due to the co-expression between p110α and mTOR seen in our clinical specimens, we studied synergism between the mTOR inbitor rapamycin and NVP-BKM120 and rapamycin and LY294002. Concentrations of 1000 and 500 nM of NVP-BKM120 were combined with a range of concentrations of rapamycin (1, 10 and 100 nM) in six NSCLC cell lines. Synergism was seen in five of the six cell lines at all concentrations of NVP-BKM120 with all three concentrations of rapamycin, and in the sixth cell line (H1650), synergy was seen at higher concentrations of NVP-BKM120 (**Figure 3** and **Tables S3 and S4**). We then studied synergism between LY294002 and rapamycin. Concentrations of 5000 and 2500 nM of LY294002, were combined with a range of concentrations of rapamycin (1, 10 and 100 nM) in six lung cell lines. Synergism was seen in all six cell lines at all concentrations of LY294002 with all three concentrations of rapamycin as shown in **Table S3**. **Figure 3** shows the synergism graphically, using H2170 and SW900 as an example. Notably, the differences seen in viability when adding 1 nM, 10 nM or 100 nM rapamcyin were fairly similar.

### Activity of a dual PI3K/mTOR inhibitor in NSCLC cell lines

**Figure 4:**
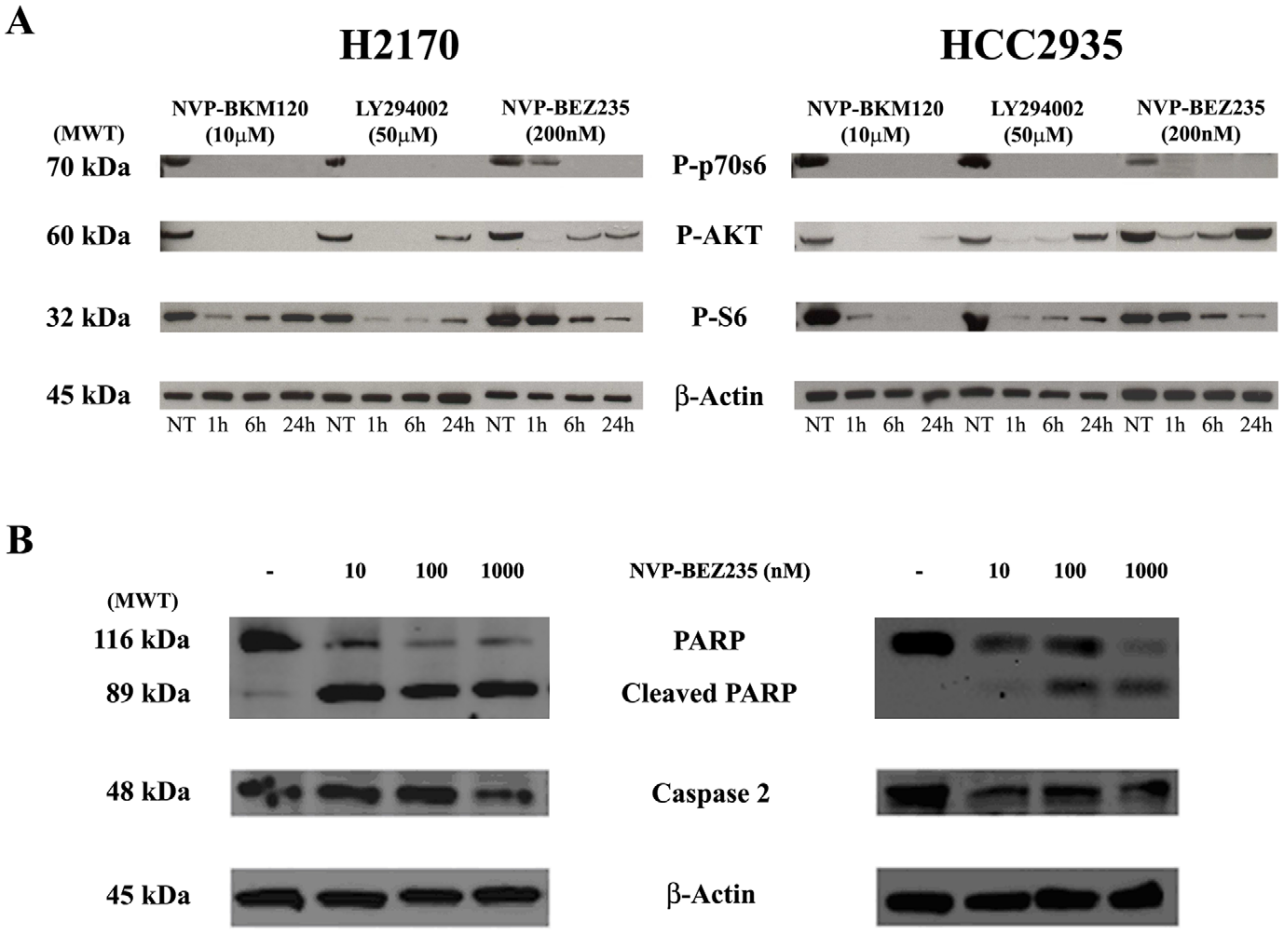
Effects of NVP-BKM120, LY294002 and NVP-BEZ235 on pAKT, pP70S6K, and pS6 expression in HCC2935 and H2170 lung cancer cell lines. **Panel A**: HCC2935 and H2170 cells were treated with the indicated concentration of NVP-BKM120, LY294002 or NVP-BEZ235 for 1, 6 or 24 hours and harvested at these time points. Cells were lysed and proteins were extracted, electrophoresed and probed for expression of phosporylated AKT (Ser^473^), phosphorylated p70S6K (Thr^389^) and phosphorylated S6 Ribosomal Protein (Ser^235/236^). Protein gel loading was assessed by expression of β-Actin. **Panel B**: Effects of NVP-BEZ235 on apoptosis in HCC2935 and H2170 lung cancer cell lines. Treatment of H2170 and HCC2935 cells with ascending concentrations of NVP-BEZ235 resulted in dose-dependent PARP cleavage and caspase-2 induction.

Given the synergism seen between PI3K inhibitors and rapamycin in lung cancer cell lines, a dual PI3K/mTOR inhibitor that has been given to solid tumor patients in phase I clinical trials, NVP-BEZ235, was studied. In all six lung cancer cell lines the IC_50_s for the NVP-BEZ235 compound were in the nM range while no effect was seen in HBE135-E6E7 cells, even at concentrations as high as 1000 nM or twenty times the average IC_50_ observed in NSCLC cell lines (**Table 1**). These results suggest that NSCLC growth and survival mediated by a broad range of molecular factors are selectively sensitive to inhibition of PI3K by NVP-BEZ235. Targets of NVP-BEZ235 (pAKT, pP70S6K and pS6) were determined by immunoblot analysis in cells with up to 24 hours drug exposure. Any drug exposure beyond 24 hours resulted in significant amount of dead cells and debris and would have limited the interpretability. pAKT, pP70S6K and pS6 decreased with exposure to the drug in a time-dependent fashion, as shown by immunoblot analysis in **Figure 4** **, panel A** for H2170 and HCC2935 cell lines. Relative to untreated cells, pAKT, pP70S6K, and pS6 were down-regulated with NVP-BKM120, LY294002, and NVP-BEZ235.

NVP-BEZ235 was previously shown to cause PARP cleavage and induce apoptosis through activation of caspase-2, but not caspases-8, -9, and -10 [34]. We therefore studied the effect of NVP-BEZ235 on PARP cleavage and caspase-2 activation in two most sensitive lung cancer cell lines, HCC2935 and H2170. As shown in **Figure 4** **, panel B** NVP-BEZ235 treatment results in PARP cleavage and modest caspase-2 activation. Cleaved caspase-2 was not detected by western blotting in this assay.

### Synergism between the dual PI3K/mTOR inhibitor NVP-BEZ235 and the EGFR inhibitor erlotinib in NSCLC cell lines

**Figure 5:**
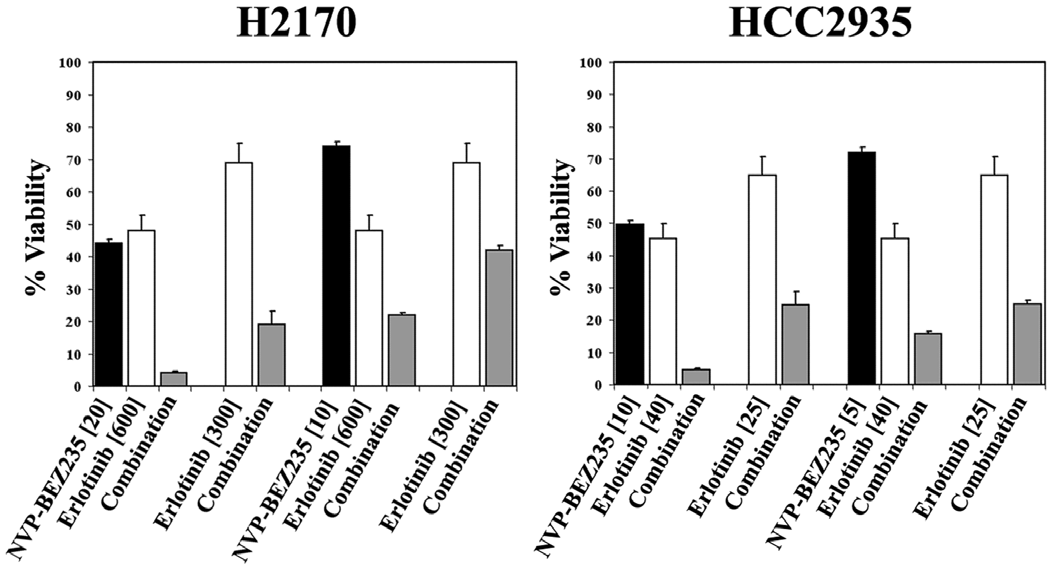
Combinations of either of NVP-BEZ235 and Erlotinib in H2170 (left) and HCC2935 (right) lung cancer cell lines. H2170 and HCC2935 cells were treated with the PI3K inhibitor NVP-BEZ235 alone or combined with Erlotinib at their respective single drug nanomolar IC_50_ or IC_25_ concentrations. Viability was measured at 72 hours with the Cell Titer Glo assay. The bars show viability as a percentage of viable cells relative to untreated cells. Three separate experiments were performed and each condition was measured in three replicate wells.

Our results so far show that mTOR blockade is necessary to enhance the inhibition of the PI3K pathway. Given that inhibiting only one or two steps of the survival and proliferation pathways often proves to be therapeutically insufficient due to the various escape mechanisms, we studied the efficacy of multi-level targeting by adding EGFR tyrosine kinase inhibition to dual PI3K/mTOR inhibition in the same set of six NSCLC cell lines. Cells were treated with the PI3K inhibitor NVP-BEZ235 alone or combined with erlotinib, at their respective single drug IC_50,_ IC_25_ or IC_10_ concentrations (**Table 1** **and Tables S5 and S6**), and viability was measured at 72 hours with the Cell Titer Glo assay. As shown in **Table S6**, synergism was seen in all six cell lines studied, including SW900 and SK-MES-1 which are highly resistant to erlotinib alone. Notably, in all four erlotinib sensitive cell lines (HCC827, HCC2935, H2170 and H1650) synergy was relatively more enhanced at higher concentrations of erlotinib (**Table S6**). A graphic example of the combined effect of these two drugs in H2170 and HCC2935 is shown in **Figure 5** **and Table S7**. Of note, the majority of erlotinib concentrations used in these experiments are well below the reported steady-state level of erlotinib achieved in actual patients which has been reported as 1200+/−620 ng/ml [35]. This translates into 2797.2+/−1445.2 nM of erlotinib.

## Discussion

Deregulation of the PI3K/AKT/mTOR signaling pathway has been demonstrated in NSCLC. In this study, we assessed the expression of PI3K subunits p85 and p110α in NSCLC tumor specimens. In the two cohorts we studied there was higher expression of p85 in adenocarcinoma compared to squamous cell carcinoma across all stages. High p85 expression was prognostic of decreased survival on univariate analysis, but not on multivariate analysis, presumably due to the strong association with disease stage. Mutations or copy number gains of *PIK3CA*, the gene encoding p110α, have been described in lung cancer, and a limitation of our study is that the *PIK3CA* genetic status is unknown in the tumor specimens of our cohorts. However, the expression of p85 might be a more valuable indicator to study, as several studies have demonstrated that p110α that is expressed in excess of p85 is unstable and rapidly degraded when not bound to p85 [36]–[38]. Immunohistochemical staining of tumor specimens for p110α is therefore likely going to underestimate the impact of p110α and changes in *PIK3CA* status. Furthermore, p85 has been proposed to have a regulatory function by associating with proteins other than p110 α such as IRS-1 and PTEN [38], [39]. Even tumor suppressor properties of p85 have been proposed based on observations in mice with a liver-specific deletion of the PiK3r1 gene [40]. We also cannot rule out the possibility that other isoforms of the catalytic subunit such as p110β, p110δ and p110γ might be involved in NSCLC as it has been described in other cancers. For instance, a recent study suggested a critical role for p110γ in pancreatic cancer [41].

Our findings are consistent with previous observations in a smaller cohort that included 73 cases of primary NSCLC which indicated that high p85 expression was associated with higher tumor grade and metastatic disease [26]. By using a quantitative method (AQUA), we were able to confirm the correlation between high p85 expression and poor survival and higher stage in a large independent cohort of NSCLC patients.

The association between high p85 expression and disease aggression demonstrated by our data and corroborated by results published by other investigators pointing to the role of PI3K in cancer, suggests that PI3K might be a valuable therapeutic target in NSCLC and warrants further investigation using novel and more effective PI3K inhibitors, such as those studied here. However, resistance to PI3K inhibition has been attributed to numerous mechanisms including negative feedback loops. One of the events downstream of PI3K activation is the activation of AKT through phosphorylation on Thr^308^ and Ser ^473^. Increasing evidence has emerged that a rapamycin-insensitive mTOR complex is the kinase responsible for AKT activation resulting from phosphorylation on Ser ^473^ [42], [43] which paradoxically allows mTOR to be both upstream and downstream of itself [44]. It has been suggested that targeting the PI3K pathway at multiple sites might be required to interrupt feedback loops to achieve optimal outcomes [5]. We were able to demonstrate synergistic effects by co-targeting PI3K and mTOR in NSCLC cell lines using LY294002, a commercially available PI3K inhibitor, and NVP-BKM120, a novel clinical quality PI3K inhibitor, alone and in combination with rapamycin. This study is the first report of the effects of NVP-BKM120 in NSCLC cell lines. A recently completed phase 1 study of NVP-BKM120 included two patients with pre-treated lung cancer with one patient remaining on study for more than 8 months [45].

While our AQUA data show a higher expression of p85 and p110α in adenocarcinoma compared to squamous cell carcinoma, it does not appear in the limited number of cell lines that were studied that adenocarcinoma cell lines are more sensitive to PI3K inhibitors than squamous carcinoma cells.

m-TOR inhibitors like rapamycin and its analogues (rapalogs) have cytostatic properties in preclinical models [46]. However, these drugs have had only limited activity when administered alone to patients with NSCLC, presumably because they interrupt negative feedback loops that down-regulate PI3K signaling, causing paradoxical up-regulation of pro-survival signaling pathways. Our data suggest that rapalogs in combination with a PI3K inhibitor may limit this up-regulation and could act as sensitizers to direct PI3K inhibition in NSCLC. This finding is consistent with previous reports of activity by combining PI3K and mTOR inhibitors in various types of cancer cells [47], [48]. Our observation that minimal mTOR inhibition is sufficient to achieve synergism with direct PI3K inhibitors is very important, as this may translate into better clinical tolerability without sacrificing efficacy of this drug combination.

Dual inhibitors of PI3K and mTOR have demonstrated promising activity in a number of malignancies [34], [48], [49]. NVP-BEZ235, a novel dual inhibitor of PI3K and mTOR, was highly active in all NSCLC cell lines tested with IC_50_s in the nanomolar range and led to downregulation of pAKT and pP70S6K. This result is consistent with the effects of NVP-BEZ235 in NSCLC cell lines recently published by other investigators [50]–[52]. In addition, we were able to demonstrate that NVP-BEZ235 resulted in PARP cleavage and caspase-2 activation. This is consistent with previous studies demonstrating that NVP-BEZ235 induced apoptosis through activation of caspase-2 but not caspases-8, -9 and -10 [34], [48]. Our results with NVP-BEZ235 are consistent with previous studies showing antiproliferative effects of NVP-BEZ235 in a transgenic mouse model of lung cancer [25]. NVP-BEZ235 has now entered early phase clinical trials for solid tumors. Five patients with lung cancer were included in a recently completed Phase I study with two of them demonstrating response by CT and PET criteria to NVP-BEZ325 [53].

In the limited number of lung cancer cell lines that we studied the response to co-inhibition by PI3K inhibitors and rapamycin or to the dual inhibitor, NVP-BEZ235, was independent of wild-type or mutant EGFR status. This observation differs somewhat from a previous report by Faber et al. [54] describing insufficient antiproliferative effects of NVP-BEZ235 in the EGFR mutant NSCLC cell line HCC827. However, it has to be noted that HCC827 – unlike the other cell lines with EGFR mutations – was also the cell line least sensitive to NVP-BEZ235 in our hands.

We explored the possibility of targeting the PI3K/AKT pathway at multiple levels by adding the direct EGFR tyrosine kinase inhibitor erlotinib to the dual inhibition of PI3K and mTOR by NVP-BEZ235. Erlotinib is approved by the FDA as single agent therapy for NSCLC after first and second line chemotherapy [55], however the response rates are low at an average of 8.9% and the median duration of response is modest at 7.9 months. While we observed a wide range of IC_50_s for erlotinib which spanned four logs between the most sensitive and the most resistant cell lines, erlotinib potentiated the growth inhibition by NVP-BEZ235 in all cell lines studied. These results support a potential therapeutic role of co-targeting of EGFR and the PI3K pathway and suggest that this approach should be evaluated further for patients with NSCLC. Of note, the concentrations of erlotinib used in majority of these experiments were well below the reported steady state level of erlotinib achieved in actual patients [35].

In summary, here we showed that PI3K expression is associated with advanced stage and decreased survival in NSCLC, suggesting that it might be a good drug target for this disease. The p110α subunit was strongly co-expressed with mTOR. Concurrent inhibition of PI3K and mTOR was synergistic in all NSCLC cell lines studied and resulted in growth inhibition and apoptosis. It appeared that minimal amount of rapamycin in the nanomolar range was sufficient to potentiate the effect of the PI3K inhibitors, LY294002 and NVP-BKM-120. This could potentially translate into decreased toxicity and better clinical tolerability of the drug combination. Dual inhibition of PI3K and mTOR by NVP-BEZ-235 is promising and should be further evaluated in clinical trials for patients with NSCLC alone and in combination with EGFR inhibitors.

## Supporting Information

Figure S1 **Expression of PI3K (p85 or p110α) and total and phosphorylated AKT in NSCLC lysates.** Pretreatment PI3K, AKT and phosphorylated AKT levels were variable among cell lines and there is no apparent association between these levels and sensitivity/resistance to NVP-BKM120 or LY294002.
(TIF)

Table S1 **Characteristics of cell lines.** References for mutational status of cell lines: http://www.sanger.ac.uk/perl/genetics/CGP/cosmic/ http://www.atcc.org.
(XLS)

Table S2 **AQUA Score Distribution.**
(XLS)

Table S3 **Combination Indices (CI) for NVP-BKM120 and rapamycin or LY294002 and rapamycin in six lung cancer cell lines.** (Synergistic combinations are highlighted in blue.)
(XLS)

Table S4 **Viability data for NVP-BKM120 and rapamycin or LY294002 and rapamycin in H2170 and SW900 lung cancer cell lines.**
(XLS)

Table S5 **IC_50_ of Erlotinib in a panel of six lung cancer cell lines.**
(XLS)

Table S6 **Combination Indices (CI) for NVP-BEZ235 and Erlotinib in six lung cancer cell lines.** (Synergistic combinations are highlighted in blue.)
(XLS)

Table S7 **Viability data for NVP-BEZ235 and Erlotinib in H2170 and HCC2935 cancer cell lines.**
(XLS)
